# Nutritional status and associated factors among people living with HIV/AIDS in Ghana: cross-sectional study of highly active antiretroviral therapy clients 

**DOI:** 10.1186/s40795-021-00418-2

**Published:** 2021-05-27

**Authors:** Beauty Mawuenam Nanewortor, Farrukh Ishaque Saah, Prince Kubi Appiah, Hubert Amu, Kwaku Kissah-Korsah

**Affiliations:** 1 Department of Family and Community Health, School of Public Health, University of Health and Allied Sciences, Hohoe, Ghana; 2 Department of Epidemiology and Biostatistics, School of Public Health, University of Health and Allied Sciences, Hohoe, Ghana; 3 Department of Population and Behavioural Sciences, School of Public Health, University of Health and Allied Sciences, Hohoe, Ghana; 4 Department of Population and Health, University of Cape Coast, Cape Coast, Ghana

**Keywords:** Ghana, HIV/AIDS, Nutritional status, People living with HIV/AIDS, Nutritional knowledge

## Abstract

**Background:**

Nutrition among people living with HIV/AIDS (PLWHA) is essential in their care and management as it has implication for their immune function. We examined the nutritional status and associated factors among HIV positive clients accessing Highly Active Anti-Retroviral Therapy (HAART) at a public hospital in Ghana.

**Methods:**

This was a descriptive cross-sectional study of 152 clients. Anthropometric measurements for weight and height were carried out in 2019. Data were analysed using SPSS 22.0. Descriptive and analytical statistics comprising frequency, percentage, and binary logistic regression were adopted in presenting the results.

**Results:**

Seventy-nine percent and 74% of the clients had good nutrition knowledge and attitude, respectively. Also, 42% were malnourished (underweight = 13.8%, and overweight = 28.3%). Clients with primary (AOR = 0.36, 95% CI = 0.07–1.84), JSS/JHS (AOR = 0.26, 95% CI = 0.08–0.84), SSS/SHS (AOR = 0.22, 95% CI = 0.05–1.02) and tertiary (AOR = 0.26, 95% CI = 0.08–0.88) were less likely to be malnourished compared with those with no formal education. Those with good nutrition-related knowledge were 56% (AOR = 0.44, 95% CI = 0.18–1.09) less likely to be malnourished than those with poor knowledge; this was however, not statistically significant.

**Conclusion:**

We found a high prevalence of malnutrition among the clients which militates against progress towards achieving the Sustainable Development Goal targets 3.3 and 3.4 of stopping AIDS epidemic and preventing premature deaths from malnutrition. Our findings justify the need for the implementation of innovative interventions by stakeholders in Ghana’s health industry to improve the nutritional status of people living with the disease.

**Supplementary Information:**

The online version contains supplementary material available at 10.1186/s40795-021-00418-2.

## Introduction

The Sustainable Development Goals (SDGs) promulgated by the United Nations in the year 2015 seek to improve health for all at all ages in goal three [1]. Inherent in this goal are targets 3.3 and 3.4, which seek to end the Human Immune Virus and Acquired Immune-deficiency Syndrome (HIV/AIDS) epidemic and reduce premature mortality from non-communicable diseases, including malnutrition [2]. However, these conditions continue to be critical public health issues globally. In 2019, it was estimated that globally, 38 million people are living with HIV/AIDS, with 67% of those who were aware of their status on antiretroviral treatment [3].

The AIDS stages of the condition make the immune system vulnerable to opportunistic infections with common symptoms being weight loss, fever, diarrhoea, and cough [4]. Poor nutrition, however, reduces the immunity of people living with HIV/AIDS (PLWHA) and results in a quicker progression to the AIDS stage [5, 6]. An immune dysfunction as a result of HIV/AIDS leads to malnutrition, while poor nutrition decreases the immune system [7]. Undernutrition among PLWHA is known to be caused by depression, reduced appetite, and some common opportunistic infection such as oral thrush.

Good nutrition primes to stronger resistance to the disease, upsurges compliance to the efficacy of antiretroviral treatment, promotes a higher quality of life, protects the organism from immunosuppression, and adjourns the stage of AIDS [5, 6]. Also, it is argued that good nutrition among PLWHA helps to enhance the immune system and decrease the burden of opportunistic infections [8–11].

Three or more antiretroviral drugs have been the standard treatment for HIV/AIDS, which represses viral replication. It is termed the Highly Active Antiretroviral Therapy (HAART) [12]. In 2013, WHO reported that the antiretroviral treatment was accessible to some 12.9 million folks, the treatment significantly enhances the life expectancy and health-related quality of life [12]. HAART in itself influences the normal metabolic processes, therefore affecting the nutritional intake of PLWHA. Hence, nutrition and diet are predominantly significant in the use of the therapy [5]. Understanding nutritional requirements and proper nutritional practices among PLWHA is critical to improving their immune system. A meta-analysis of demographic and health survey data of women in reproductive age from 11 Sub-Saharan African countries including Ghana, for instance showed 10% had HIV/AIDS-related malnutrition (BMI < 18.5) [13].

In Ghana, HIV prevalence continues to rise with a national prevalence of 1.70% in 2019 with Volta Region recording a prevalence of 1.28% [14]. There have been significant achievements in the care, treatment, and support for PLWHA in Ghana since HAART became accessible [15]. However, Nti, Hayford, and Opare-Obisaw [16] note that one-third of PLWHA in the Eastern Region of Ghana were underweight while 50% had poor diets. Also, most of the 50 HIV-seropositive adults attending a hospital in Accra, had inadequate dietary intake citing cost and knowledge of nutritional requirements as barriers [17]. If services to preserve and prolong the lives of PLWHA, including better nutritional practices are not implemented, attaining projected results will not be possible. We, therefore, investigated the nutrition-related knowledge and attitude, and nutritional status of PLWHA accessing HAART at a public hospital in the Volta Region of Ghana. Our study is relevant in the prevention of HIV/AIDS-related mortalities, in that, it provides the magnitude of poor nutrition and existing knowledge and attitudes among PLWHA in Ghana. These findings will be significant to policies and interventions to improve survival rates and quality of life for PLWHA in the country.

## Methods

The methods were carried out in accordance with relevant guidelines and regulations in this section. We adopted the “Strengthening the Reporting of Observational Studies in Epidemiology” (STROBE) checklist in conducting and reporting this research.

### Setting

The study was carried out at a public hospital in Ho Municipality of the Volta Region, Ghana in 2019. Ho has four hospitals and three clinics with the Ho Teaching Hospital, popularly known as Trafalgar, being the general and referral centre [18]. The hospital has a HAART unit where PLWHA usually visits monthly to access their immunity and medications. The unit has a counselling section that usually takes up the mandate of advising clients on their upkeep. The Municipality has a central market and three tertiary institutions.

### Study design

We adopted a descriptive cross-sectional design in conducting this study. This design allowed to collect quantitative data at a point in time using questionnaires to describe an existing phenomenon of interest, nutritional status. It was appropriate because the study sought to explore nutrition-related knowledge, attitudes, and nutritional status of PLWHA accessing HAART in a public hospital in Ghana.

### Study population and sampling

The study involved people living with HIV/AIDS, accessing HAART at a public hospital, and aged 18 years or older. This population was chosen because they are experiencing service that is meant to impact the phenomena of this study; nutritional knowledge and practices among PLWHA. However, health professionals and foreign nationals were excluded.

The study utilised Yamane’s [19] sample size formula, $n=\frac{\mathrm{N}}{1+\mathrm{N}{\left(\mathrm{e}\right)}^2}$ to determine the sample sizes based on the population of PLWHA accessing HAART at the facility, 210. At a 95% confidence interval, margin of error, e = 0.05. Thus, with a 10% non-response rate, the study used a sample size of 152 PLWHA attending HAART at the public hospital. These respondents were selected using a simple random sampling technique. Using the HAART Register as a sampling frame, the numbers corresponding to each client in the register were written on pieces of paper, rolled into balls, and put into a container. The container was shaken and a rolled paper picked at random until the sample size was obtained. Clients whose numbers were picked were included in the study to make up the required sample.

### Procedures

A person living with HIV/AIDS whose registration number was picked, and consented to participate was interviewed by a trained research assistant. Prior to inclusion in the study, the study processes were explained and assurance of ethical concerns and all other concerns addressed. Data were collected using a semi-structured questionnaire with support of two trained research assistants. The instrument was self-developed based on literature (largely adapted form Abgaryan [5]) and pre-tested among 40 PLWHA in the Hohoe Municipal Hospital and found to have a reliability coefficient, ∝ of 0.742. Due to the differences in contexts of the previous studies and this study, the validity of the instrument was ensured by giving it to two experts in HIV/AIDS studies to review which resulted in some parts being discarded or remodelled for use. It was made up of four sections A-D; Section A on socio-demographic characteristics, B on nutrition-related knowledge and healthy diet, C on the attitude of PLWHA towards healthy nutrition, and D on nutritional status. Respondents who could not read or understand the questionnaire were assisted, and the questions explained to them. To minimize the introduction of bias in the translation, the data collectors were trained on common explanation to all the questions. Completed questionnaires were checked for completeness and validity of responses.

### Study variables

The outcome variable was nutritional status, which was determined using the Body Mass Index (BMI). The explanatory variables were age, sex, marital status, highest educational level, employment status, size of the household, monthly expenditures, as well as the proportion of expenditure spent on food, nutrition-related knowledge and attitude towards nutrition. The nutrition-related knowledge included the understanding of the nutritional requirements for PLWHA and the need for special nutrition while attitude towards nutrition constituted questions assessing behaviour and perception of special nutrition for PLWHA.

### Data analysis

Data collected were entered, cleaned, and analysed using Statistical Package for the Social Sciences (SPSS) version 22.0. The analyses were carried out with descriptive statistics comprising mean, frequency, and percentage, as well as inferential statistics consisting of binary logistic regression. All statistical analyses were considered significant at *p*-values < 0.05 at 95% confidence intervals (CI). We performed two binary logistics regression models. We first conducted bivariable model (Model I) using all the explanatory variables. Variables that were significant were then included in our multivariable model (Model II).

There were 20 questions assessing nutrition-related knowledge, and correct responses were graded 1 and wrong responses scored 0, resulting in a maximum total score of 20. Hence, respondents who had index scores of more than half i.e. 11 or more (indicating correct responses for more than 10 of the 20 knowledge questions) were categorized as having adequate nutrition-related knowledge. The attitude variable constituted five [5] questions and used a Likert scale of 0-Disagree and 1-Agree to grade, making a maximum index score of 5. Thus, respondents with attitude scores of more than half i.e. 3 or more (representing positive responses for 3 or more of the 5 attitude statements) were graded as having good attitude towards their nutrition.

### Ethical issues

Ethical approval for this study was obtained from the University of Health and Allied Sciences’ Research Ethics Committee. Permission was also sought from the Ho Municipal Health Directorate and the management of the public hospital. Before including participants in the study, written informed consent was obtained, after assurance of optimum levels of anonymity and confidentiality of information provided. Hard copies of the data have been kept under lock and key, while soft copies have been stored on a personal computer with password and backup.

## Results

### Socio-demographic characteristics

Table 1 presents the socio-demographic characteristics of the 152 HIV/AIDS clients attending antiretroviral therapy at the public hospital. Females constituted 77.6%. The mean age of the respondents was 39.3 years (S.D. = 12.6). A majority (54.6%) were married, Christians (86.8%), and Ewes (77%). The highest level of education attained by the comparative majority of the respondents (42.1%) was primary education. Most also lived outside the Ho Municipality (66.4%) and were employed (86.8%). Also, 34.9% of the respondents were engaged in trading as their major occupation. A comparative majority of the respondents (32.2%) has been on antiretroviral therapy for 5–10 years. Most (97.4%) of the respondents had also received nutritional counselling.

**Table 1:** Socio-demographic characteristics of respondents

Socio-demographic variable	Frequency	Percentage (%)
**Sex**		
Male	34	22.4
Female	118	77.6
**Age (in completed years)**		
< 20	6	3.9
20–29	32	21.1
30–39	46	30.3
40–49	32	21.1
50–59	27	17.8
60+	9	5.9
**Marital status**		
Never married	32	21.1
Married	83	54.6
Divorced	19	12.5
Widowed	18	11.8
**Religion**		
Christian	132	86.8
Muslim	17	11.2
Traditionalist	3	2.0
**Ethnicity**		
Akan	23	15.1
Ewe	11	77.0
Ga	10	6.6
Mole-Dagbani	2	1.3
**Highest educational level**		
No formal education	10	6.6
Primary	64	42.1
JSS/JHS	14	9.2
SSS/SHS	46	30.3
Tertiary	18	11.8
**Place of residence**		
In Ho Municipality	51	33.6
Outside Ho Municipality	101	66.4
**Employment status**		
Employed	132	86.8
Unemployed	20	13.2
**Occupation (*n*** **= 132)**		
Artisan/Skilled worker	41	31.1
Trader	53	40.2
Farmer	19	14.4
Teacher	7	5.3
Retired	1	0.8
Other	11	8.3
**Length of being on anti-retroviral therapy**		
≤ 6 months	13	8.6
7–11 months	10	6.6
1–2 years	32	21.0
3–4 years	45	29.6
5–10 years	49	32.2
> 10 years	3	2.0
**Received nutritional counselling**		
Yes	148	97.4
No	4	2.6

### Nutrition-related knowledge and attitude among PLWHA

Participants’ responses to the knowledge and attitude questions is attached as Additional File 1. The nutrition-related knowledge questions encompassed; expected consumption rates (more, less or the same) for vegetables, grains, meat, and fruits, and sugary, salty, and fatty foods, as well as knowing experts’ nutrition recommendation for PLWHA, inter-related link between nutrition and health status of PLWHA.

Table 2 presents the overall level of nutrition-related knowledge among the HIV/AIDS clients. It was realised that, overall, 78.9% of the respondents had good nutrition-related knowledge. Regarding attitude towards nutrition among the respondents, the questions included perception of maintaining healthy diet, willingness to make diet changes, and perception of healthy diet education. We realised that 74% of the respondents had good attitude towards their nutrition.

**Table 2:** Level of nutrition-related knowledge and attitude of respondents

Variable	Frequency	Percentage (%)
**Level of knowledge**		
Poor knowledge	32	21.1
Good knowledge	120	78.9
**Nutritional attitude**		
Poor attitude	39	26.0
Good attitude	113	74.0

### Nutritional status of PLWHA

**Fig. 1:**
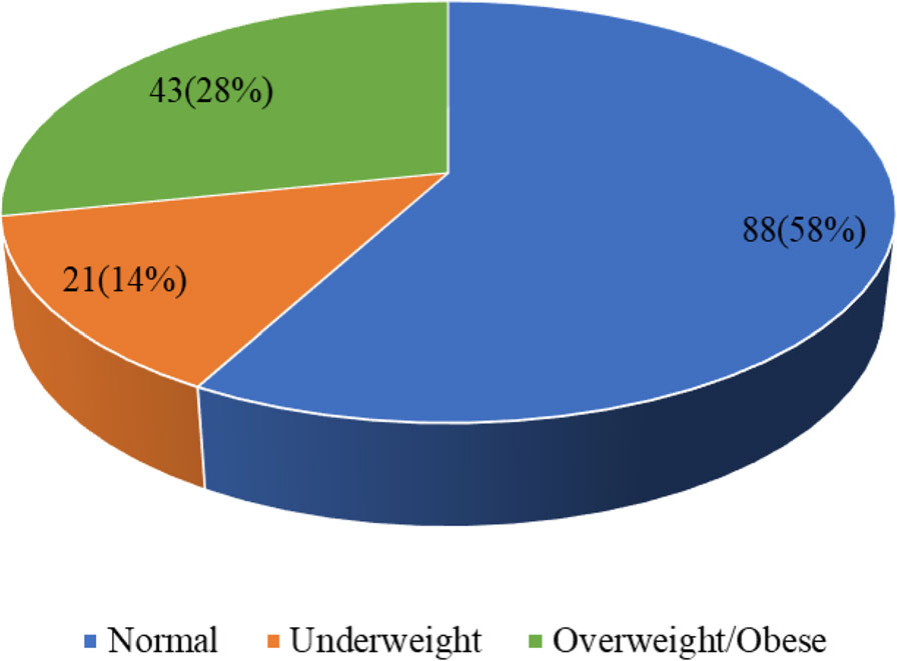
Nutritional status of HIV/AIDS clients

The nutritional status of the respondents was determined using the weights and height of the respondents and categorised into Normal and Malnourished (underweight and overweight/obese). From Fig. 1, 14% of the respondents were underweight, whereas 28% were overweight/obese. Thus, 42% of the respondents were malnourished (either underweight or overweight/obese).

### Predictors of nutritional status among PLWHA

Our result in Table 3 shows that PLWHA with primary (COR = 0.38, 95% CI = 0.08–1.93, *p* = 0.245), JSS/JHS (COR = 0.2, 95% CI = 0.07–0.73, *p* = 0.012), SSS/SHS (COR = 0.21, 95% CI = 0.05–0.96, *p* = 0.044), and tertiary (COR = 0.22, 95% CI = 0.07–0.74, *p* = 0.014) were less likely to be malnourished compared with those without formal education. Also, PLWHA who had good nutrition-related knowledge were 62% (COR = 0.38, 95% CI = 0.16–0.92, *p* = 0.031) less likely to be malnourished than those with poor nutrition-related knowledge.

**Table 3:** Predictors of Nutritional Status among PLWHA

Variable	Nutritional status		Model I	Model II
	Malnourished n(%)	Normal n(%)	COR (95%CI) ***p***-value	AOR (95%CI) ***p***-value
**Sex**				
Male	12(35.3)	22(64.7)	Ref	
Female	52(44.1)	66(55.9)	0.69 (0.31-1.53) 0.363	
**Age group**				
<20	3(50.0)	3(50.0)	Ref	
20-29	13(40.6)	19(59.4)	2.00 (0.24-16.61) 0.521	
30-39	20(43.5)	26(56.5)	1.37 (0.29-6.48) 0.693	
40-49	15(46.1)	17(53.1)	1.54 (0.34-6.92) 0.574	
50-59	10(37.0)	17(63.0)	1.76 (0.37-8.32) 0.473	
60+	3(33.3)	6(66.7)	1.18 (0.24-5.77) 0.841	
**Marital status**				
Single	14(43.8)	18(56.3)	Ref	
Married	34(41.0)	49(59.0)	0.97 (0.30-3.11) 0.962	
Divorced	8(42.1)	11(57.9)	0.87 (0.31-2.42) 0.786	
Widowed	64(42.1)	10(55.6)	0.91 (0.25-3.34) 0.886	
**Religion**				
Christian	57(43.2)	75(56.8)	Ref	
Muslim	6(35.3)	11(64.7)	1.52 (0.13-17.18) 0.735	
Traditionalist	1(33.3)	2(66.7)	1.09 (0.08-14.66) 0.948	
**Ethnicity**				
Akan	13(56.5)	10(43.5)	Ref	
Ewe	45(38.5)	72(61.5)	1.30 (0.07-23.43) 0.859	
Ga	5(50.5)	5(50.5)	0.62 (0.04-10.24) 0.742	
Mole-Dagbani	1(50.0)	1(50.0)	1.00 (0.05-20.83) 1.000	
**Highest level of education**				
No formal education	5(50.5)	5(50.5)	Ref	Ref
Primary	24(37.5)	40(62.5)	0.38 (0.08-1.93) 0.245	0.36 (0.07-1.84) 0.222
JSS/JHS	5(35.7)	9(64.3)	0.23 (0.07-0.73) 0.012*	0.26 (0.08-0.84) 0.025*
SSS/SHS	17(37.0)	29(63.0)	0.21 (0.05-0.96) 0.044*	0.22 (0.05-1.02) 0.053
Tertiary	13(72.2)	5(27.8)	0.22 (0.07-0.74) 0.014*	0.26 (0.08-0.88) 0.031*
**Place of Permanent living**				
Inside Ho Municipality	20(39.2)	31(60.8)	Ref	
Outside Ho Municipality	44(43.6)	57(56.4)	0.84 (0.42-1.66) 0.608	
**Employment status**				
Yes	53(40.2)	79(59.8)	Ref	
No	11(55.0)	9(45.0)	0.55 (0.21-1.41) 0.215	
**Duration of being on therapy**				
Less than 6 months	6(46.2)	7(53.8)	Ref	
7-11 months	5(50.0)	5(50.0)	1.71 (0.12-23.94) 0.689	
1-2 years	13(40.6)	19(59.4)	2.00 (0.13-29.81) 0.615	
3-4 years	20(44.4)	25(55.6)	1.34 (0.11-16.70) 0.806	
5-10 years	19(38.8)	30(61.2)	1.60 (0.13-18.94) 0.709	
More than 10 years	1(33.3)	2(66.7)	1.27 (0.11-14.95) 0.851	
**Have you received nutritional counselling**				
Yes	62(41.9)	86(58.1)	Ref	
No	2(50.0)	2(50.0)	0.72 (0.0-5.26) 0.747	
**Nutrition-related knowledge**				
Poor Knowledge	8(25.0)	24(75.0)	Ref	Ref.
Good Knowledge	56(46.7)	64(53.3)	0.38 (0.16-0.92) 0.031*	0.44 (0.18-1.09) 0.076
**Attitude towards nutrition**				
Poor attitude	17(43.6)	22(56.4)	Ref	
Good attitude	47(41.6)	66(58.40	1.08 (0.52-2.26) 0.828	

*p<0.05 *Ref.* Reference category

After adjusting for presence of other significant variables, we found that PLWHA with primary (AOR = 0.36, 95% CI = 0.07–1.84, *p* = 0.222), JSS/JHS (AOR = 0.26, 95% CI = 0.08–0.84, *p* = 0.025), SSS/SHS (AOR = 0.22, 95% CI = 0.05–1.02, *p* = 0.053) and tertiary (AOR = 0.26, 95% CI = 0.08–0.88, *p* = 0.031) were less likely to be malnourished compared with those without formal education.

Also, PLWHA who have good nutrition-related knowledge were 56% (AOR = 0.44, 95% CI = 0.18–1.09, *p* = 0.076) less likely to be malnourished than those who had poor nutrition-related knowledge.

## Discussion

We examined the nutrition-related knowledge and attitude, and status of HIV/AIDS clients accessing HAART service in a public hospital in the Volta Region of Ghana. We found that most of the respondents (78.9%) had good nutrition-related knowledge. The finding that most of the PLWHA had good nutrition-related knowledge is in congruence with that of Anand and Puri [20], where overall nutrition-related knowledge was found to be good among 80% of PLWHA. However, this finding differs from those of Devika and Thahira [21] and Abgaryan [5] which reported lower proportions of PLWHA, 48%, and 68.8% respectively, having good nutrition-related knowledge. The high proportion of good nutrition-related knowledge in our study could have resulted from nutritional counselling and education provided during the HAART clinic sessions, a common component in HAART service.

We also found that nutritional attitude was good among most of the respondents (74%). Regarding most of PLWHA having a good attitude towards their nutrition, this is consistent with Abgaryan’s [5] finding in a similar study where 71.2% of PLWHA had good attitude towards nutrition. Nevertheless, this finding disagrees with those of Anand and Puri [20] and Nzeagwu and Uwaegbute [22], who noted that less than half of PLWHA, 38%, and 49.2% respectively, had good attitude towards their nutrition. The finding of the current study could be attributed to the appropriate understanding obtained during the HAART clinic sessions. Nutrition counselling has been argued to bring about behaviour change [20]. Due to this, PLWHA are willing and supportive of nutrition improvement measures.

Also, we found that 13.8% of the respondents were underweight, whereas a quarter (28.3%) were overweight/obese. Generally, 42% of the respondents were malnourished and that respondents’ level of education and level of nutrition-related knowledge was found to statistically influence their nutritional status. Our finding that 13.8% were underweight is in congruence with the finding of Nzeagwu and Uwaegbute [22], which noted that 12.5% of PLWHA were underweight. However, this is lower than that of Thapa et al. [23], which revealed that one in five PLWHA was underweight. Also, the prevalence of obesity/overweight found in this study is lower than those found in the studies by Giudici, Duran, and Jaime [24] and Nzeagwu and Uwaegbute [22]. In these studies, the researchers found 34% and 36.7% respectively, were overweight/obese. Again, the overall malnutrition prevalence of 42% found in our study supports the finding of Kassa, Alle, and Tesfu [25] which reported 43% of prisoners living with HIV/AIDS being malnourished. The prevalence was however, higher than studies carried out in Gueckedou, Guinea (38%) [26], Tanzania (23%) [27] and Guinea (19.6%) [28]. Yet, the finding of the current study is lower than that of Nzeagwu and Uwaegbute [22], where 49.2% of the PLWHA were malnourished. This could be attributed to the fact that most HIV clients may have limited food choices and the opportunity to manage their nutrition due to their socio-economic status.

We also found that respondents’ highest level of education and level of nutrition-related knowledge influenced their nutritional status. This finding is in congruent to that of Thapa et al. [23], which posited that educational status significantly influence the nutritional status of PLWHA. Also, the current finding supports the findings of Nti et al. [16], that there is a positive correlation between nutrition knowledge and nutritional status among PLWHA. This may be due to knowledge providing a better understanding of the special nutritional needs of PLWHA, subsequently making it possible to engage in nutritional practices that will in turn positively strengthen their nutritional status as argued by Gaikwad et al. [29] in an Indian study.

### Strengths and limitations

This study relied on verbal reports in measuring attitude of respondents towards nutrition. There is, thus, the tendency that respondents might have provided favourable responses during the data collection.

## Conclusion

Although nutrition-related knowledge and attitude among PLWHA were good, this did not correspond to the high prevalence of malnutrition. This finding implies that most PLWHA are at risk of deteriorating immunity as a result of poor nutrition. Potentially, this may increase their risk of exposure to opportunistic infections such as tuberculosis, reducing their survival rates and quality of life. Ghana may, thus, not be able to achieve the SDG targets 3.3 and 3.4 targets of stopping the AIDS epidemic and reducing premature deaths from non-communicable conditions, including poor nutrition.

In order to forestall these findings and accelerate Ghana’s progress towards the achievement of the SDGs, the Ghana Health Services should intensify and employ innovative approaches to nutritional education and counselling for PLWHA. Also, HIV/AIDS stakeholders such as the Ghana Aids Commission and the Ghana Health Service should ensure sustained attention to the prevention, early detection, and treatment of malnutrition throughout the stages of HIV. Additionally, social support systems with a focus on assisting PLWHA should be instituted by the Ministry of Health and the Ghana Health Service to ensure that there is food security for individuals with low socio-economic status.

## Supplementary Information

**Additional file 1.**

## Data Availability

The datasets used and/or analysed during the current study are available from the corresponding author on reasonable request.

## Abbreviations

SDGs: Sustainable Development Goals
WHO: World Health Organisation
STROBE: Strengthening the Reporting of Observational Studies in Epidemiology
GSS: Ghana Statistical Service
HAAR: Highly Active Ante-Retroviral Treatment
BMI: Body Mass Index
PLWHA: People living with HIV/AIDS
