# Supplementary material for: Nutritional status and associated factors among people living with HIV/AIDS in Ghana: cross-sectional study of highly active antiretroviral therapy clients
Source: BMC Nutr. 2021 May 27;7:14. doi: 10.1186/s40795-021-00418-2 (PMC8157661; doi:10.1186/s40795-021-00418-2)
Supplement: Supplementary file 1 — Additional file 1. [file 40795_2021_418_MOESM1_ESM.docx]

**Nutritional status and associated factors among People Living with HIV/AIDS in Ghana: Cross-sectional study of Highly Active Antiretroviral Therapy Clients**

Beauty Mawuenam Nanewortor^1^, Farrukh Ishaque Saah^2*^, Prince Kubi Appiah^1^, Hubert Amu^3^ Kwaku Kissah-Korsah^4^

**ADDITIONAL FILE 1**

Appendix 1 provides the summary of responses on the questions used to assess respondents’ nutrition-related knowledge. These responses were used in generating the composite variable, “*Nutrition-related knowledge*”.

# Appendix 1: Nutrition-related Knowledge among PLHIV Knowledge variable

| **Knowledge Variable** | **Frequency** | **Percentage** |
| --- | --- | --- |
| **Health experts recommend that people should be eating** |  |  |
| More of Vegetable | 109 | 71.7 |
| Less of Meat | 59 | 38.8 |
| More of Grains | 105 | 69.0 |
| Less Sugary foods | 127 | 83.6 |
| More of Fruits | 122 | 80.2 |
| Less Salty foods | 126 | 82.9 |
| Less Fatty food | 113 | 74.3 |
| **How many serving of fruit and vegetable a day do you think experts are advising?** |  |  |
| Less than 13 servings | 55 | 36.2 |
| 13 servings or more | 97 | 63.8 |
| **Are people living with HIV likely to lose weight?** |  |  |
| No | 22 | 14.5 |
| Yes | 130 | 85.5 |
| **Can undernutrition deteriorate the health status of HIV-positive person?** |  |  |
| No | 64 | 42.1 |
| Yes | 88 | 57.9 |
| **Which of the mentioned food items are good sources of protein?** |  |  |
| Chicken | 137 | 90.1 |
| Greens | 16 | 10.5 |
| Cheese | 110 | 72.4 |
| Fruit | 25 | 16.4 |
| Beans | 139 | 91.4 |
| Potato | 135 | 88.8 |
| Margarine | 108 | 71.1 |
| **Which type of fat do experts say is more important for people to cut down on?** |  |  |
| Vegetable oil | 22 | 14.5 |
| Animal fat | 102 | 67.1 |
| Don’t know | 28 | 18.4 |

Appendix 2 provides the summary of responses on the questions used to assess respondents’ attitude towards nutrition. These responses were used in generating the composite variable, “*Attitude towards nutrition*”.

# Appendix 2: Attitude of PLHIV towards Nutrition

| **Attitude variable** | **Frequency** | **Percentage (%)** |
| --- | --- | --- |
| **It is beneficial to maintain a healthy diet for a person with HIV.** |  |  |
| Strongly agree | 52 | 34.3 |
| Agree | 94 | 61.8 |
| Disagree | 6 | 3.9 |
| **I do not need to make changes to my diet as it is healthy enough.** |  |  |
| Strongly agree | 67 | 44.1 |
| Agree | 73 | 48.0 |
| Disagree | 12 | 7.9 |
| **I would benefit from receiving more information on healthy diet for HIV positive people.** |  |  |
| Strongly agree | 70 | 46.0 |
| Agree | 77 | 50.7 |
| Disagree | 5 | 3.3 |
| **It will be difficult for me to keep a healthy diet.** |  |  |
| Strongly agree | 22 | 14.5 |
| Agree | 48 | 31.5 |
| Disagree | 55 | 36.2 |
| Strongly disagree | 27 | 17.8 |
| **I don’t feel able to change my diet to make it better.** |  |  |
| Yes | 11 | 7.2 |
| No | 141 | 92.8 |
